# Functional Neurological Disorder Overlapping Paroxysmal Kinesigenic Dyskinesia Confirmed by Genetic Diagnosis

**DOI:** 10.7759/cureus.42693

**Published:** 2023-07-30

**Authors:** Masayuki Ohira, Takashi Osada, Hiroaki Kimura, Terunori Sano, Masaki Takao

**Affiliations:** 1 Department of General Internal Medicine and Clinical Laboratory, National Center of Neurology and Psychiatry National Center Hospital, Tokyo, JPN; 2 Department of General Internal Medicine, National Center of Neurology and Psychiatry National Center Hospital, Tokyo, JPN

**Keywords:** functional motor disorder, functional neurological disorder, paroxysmal kinesigenic dyskinesia, neurology, psychiatry

## Abstract

Functional neurological disorder (FND) may mimic various kinds of neurologic diseases and may coexist with other neurologic disorders. In cases overlapped by FND, it might be challenging to distinguish symptoms induced by FND and those induced by other underlying neurological disorders, especially when patients show no positive signs indicative of FND. Here, we present the case of a patient who was genetically diagnosed with paroxysmal kinesigenic dyskinesia (PKD). However, most of the patient’s symptoms were considered to indicate FND. To our knowledge, there are no reports of FND overlapping PKD. This case illustrates the possibility that FND can coexist with and mimic symptoms of other diseases. It is necessary to rule out coexisting FND symptoms that may modify clinical presentations that cannot simply be explained by a recognized neurological disease.

## Introduction

Functional neurological disorder (FND) refers to various genuinely involuntary neurologic symptoms and signs such as seizures, weakness, and sensory disturbance, which have characteristic clinical features, as well as to the difficulty in voluntarily controlling these in line with a normal basic structure of the nervous system [1]. FND induces neurologic symptoms that cause substantial functional impairment, and its long-term prognosis is poor [2]. FND includes various subtypes such as seizure, limb weakness, gait disturbance, cognitive disorder, and dizziness [3]. The diagnostic criteria for FND in the Diagnostic and Statistical Manual of Mental Disorders. Fifth Edition, Text Revision (DSM-5-TR) include positive signs that support FND, such as Hoover’s sign, abductor sign, collapsing or give-way weakness, motor inconsistency, and tremor entrainment [1,4,5]. In patients with predominant motor involvement, known symptoms include weakness, tremors, jerking movements, dystonia, abnormal limb posturing, or gait disturbance, and these symptoms can be unilateral or bilateral [6]. Treatment of FND includes patient education, physical therapy, and cognitive behavioral therapy [6]. One factor that makes it difficult to diagnose FND is that it can overlap other structural conditions [3,7,8]. However, FND and other neurological disorders necessitate different treatments for each condition; therefore, it is crucial to identify the symptoms produced by either disease.

## Case presentation

**Figure 1:**
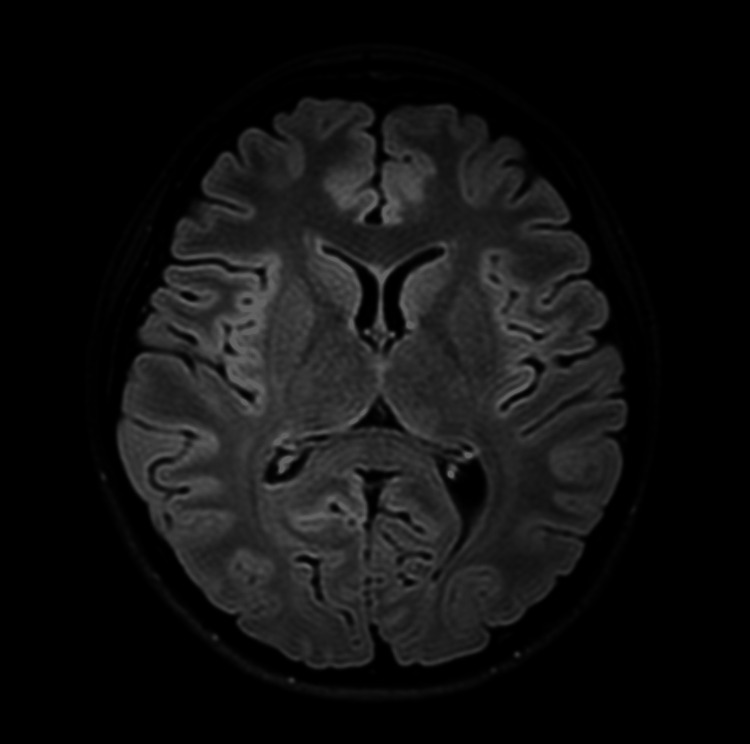
Non-contrast MRI-3T of the brain showing normal brain with no evidence of structural disease.

**Figure 2:**
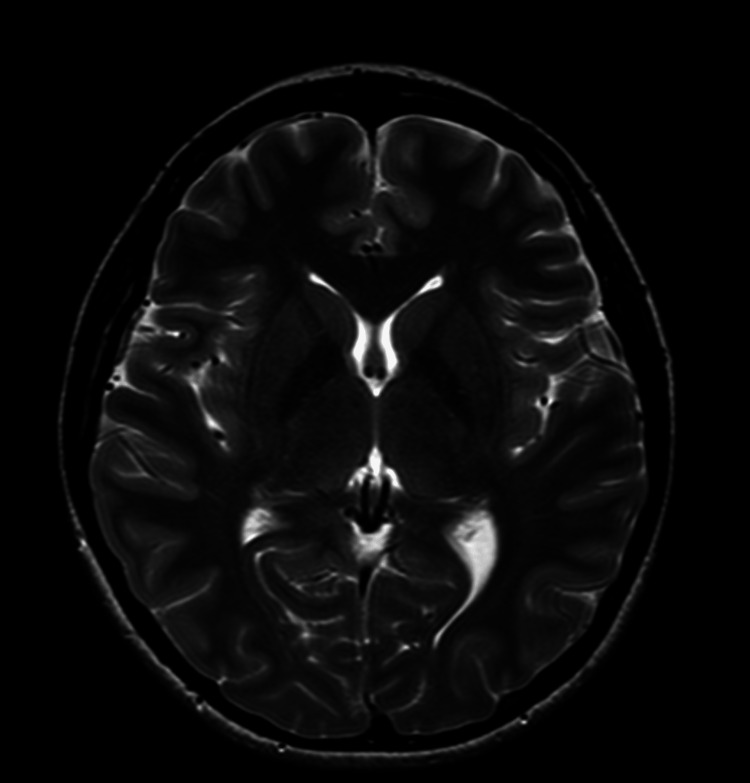
Non-contrast MRI-3T of the brain showing normal brain with no evidence of structural disease.

A 24-year-old woman presented to our hospital with involuntary movement, especially when walking. This involuntary movement was typically present in one foot and on the adduction of the ankle. This only continued for a few seconds if she made any rapid voluntary movement or tensed her muscles. This transient movement affected either leg but was not found on both legs simultaneously. There were no symptoms preceding this involuntary movement, and the patient claimed that she could not tell when the involuntary movement would occur. The patient had noticed these symptoms when she was young but had not sought medical attention because the symptoms were minimal and did not affect her daily activities. However, she had attended our clinic the previous year because she felt the symptoms had aggravated over the past few years. On her first visit to our outpatient clinic, besides the paroxysmal involuntary movement, the patient reported always having difficulty with ambulation but being able to walk unassisted despite her muscle weakness, as described below. On examination using the Medical Research Council (MRC) scale, some muscles showed weakness; the deltoid was MRC grade 4/5, biceps was MRC grade 5/5, wrist flexion MRC grade 4/5, extensor digitorum communis MRC grade 5/5, hamstrings MRC grade 5/5, and the gluteus maximus was MRC grade 2/5, without laterality. All weak muscles were affected by give-way weakness and her weak wrist flexion was compatible with the features of “paradoxical wrist flexion” [9]. All other neurological examination results were normal. The results of the MRI of the head, electromyography, and blood tests, including copper levels and creatine phosphokinase, were unremarkable (Figures 1, 2).

She had never been diagnosed with any neurological diseases. The patient only had one sibling, an older half-brother with a different mother, who had a similar clinical presentation in terms of involuntary movement, especially when he was young. No symptoms were reported by her other family members, including her father, who was common to both siblings. Based on the clinical findings and family anamnesis, we considered that the patient might have paroxysmal kinesigenic dyskinesia (PKD) in addition to FND. Genetic analysis showed a typical mutation in individuals who have PKD within the proline-rich transmembrane protein 2 (PRRT2) gene (NM_145239.2): c.649dupC (p.Arg217Profs*8) [10]. We started carbamazepine (100 mg) for involuntary movement seemingly owing to PKD, but the patient was unable to continue the medication because of sleepiness over one week. Other antiepileptic drugs such as valproic acid (100 mg) and levetiracetam (250 mg) were tried; however, the patient was unable to continue taking them owing to sleepiness, nausea, vomiting, and dizziness just a few days after she started them. Her weakness and involuntary movement continued even when she took any of these medications, and no obvious improvement was confirmed as a result of any medication. The patient continued without any medication and visited our clinic every few months for observation and to consult the psychiatrist at our hospital. However, no improvement in her symptoms has been detected so far.

After a definite diagnosis of PKD with a gene mutation in this patient, her half-brother visited our clinic for a final diagnosis of his condition. He told us that he had symptoms similar to those of his sister when he was young, but they had gradually decreased. At his first visit, he reported taking 100 mg of carbamazepine every day and having no symptoms. His neurological examination was unremarkable. A slight involuntary movement similar to that of his sister was noticeable when he did not take carbamazepine. He was finally diagnosed as having PKD with the same gene mutation as his half-sister.

## Discussion

FND has neurological symptoms that cause significant functional impairment, and these symptoms are not compatible with a currently recognized neurological or psychological disease. The DSM-5 diagnostic criteria for FND no longer require the existence of preceding stressors and instead focus on positive symptoms [4]. Thus, the DSM-5 criteria for FND include symptoms of altered voluntary motor or sensory function, clinical findings as evidence of incompatibility between the symptom and recognized conditions, symptoms that cannot be explained by another medical or mental disorder, and clinically significant distress or impairment caused by the symptoms. Some neurological disorders can trigger comorbid FND; for instance, Parkinson’s disease is more likely to precipitate FND than Alzheimer’s disease [11]. The coexistence of FND and other structural diseases can present obstacles for physicians in treatment. In these settings, recognizing the possibility that FND may overlap other diseases and considering treatment for FND when it is likely to affect symptoms is important to avoid excessive medical testing. Our patient was diagnosed with PKD based on her temporary involuntary movements and a known gene mutation related to PKD; however, her lower limb weakness was compatible with FND, supported by positive signs such as give-way weakness. Her difficulty in walking and muscle weakness were unlikely to be caused by PKD, but rather by FND. To our knowledge, there are no case reports of PKD overlapped by FND. The present case illustrates the possibility that various neurological disorders can coexist with FND, which might lead to difficulty in diagnosing other structural neurological disorders, especially those with no decisive and objective tests.

PKD is a disorder characterized by episodes of various abnormal involuntary movements typically triggered by voluntary movement. This disorder should be considered in individuals with sudden episodes of unilateral or bilateral involuntary movement and a positive family history of similar symptoms [12]. PRRT2 mutation is known to cause several diseases including PKD and c.649dupC mutation. Our patient had the most common mutation among patients with PKD, namely, PRRT2 mutation [13]. She had relatively typical features of PKD with involuntary movement and onset before 10 years of age, involuntary movement triggered by sudden voluntary movements, a short duration of several seconds, and no loss of consciousness or pain during these episodes. We were also able to diagnose her half-brother, who had the same PRRT2 mutation. He is now completely free of PKD, with minimal medication. The difference in symptoms between these siblings could indicate that our patient’s condition was mainly affected by FND.

This case illustrates typical features of both PKD and FND and the possibility that FND overlaps or can coexist across a broader range of neurological diseases than has been known until now. This report presents just one example of FND and structural diseases. Therefore, further accumulation and assessment of cases that could be accompanied by FND are necessary because the treatment could differ according to whether FND affects symptoms.

## Conclusions

Our patient presented with FND and was genetically diagnosed with PKD. We could not find any cases in the literature describing patients with PKD accompanied by FND. It is important for physicians to consider the possibility that FND can coexist with other structural diseases, especially when the latter cannot explain all symptoms. Further studies would be beneficial in determining the prevalence of unrecognized FND overlapping other diseases.
